# Two Distinct Plastid Genome Configurations and Unprecedented Intraspecies Length Variation in the *accD* Coding Region in *Medicago truncatula*

**DOI:** 10.1093/dnares/dsu007

**Published:** 2014-03-17

**Authors:** Csanad Gurdon, Pal Maliga

**Affiliations:** Waksman Institute of Microbiology, Rutgers, The State University of New Jersey, 190 Frelinghuysen Road, Piscataway, NJ 08854-8020, USA

**Keywords:** *accD*, *Medicago truncatula*, plastid genome, ptDNA, *ycf1*

## Abstract

We fully sequenced four and partially sequenced six additional plastid genomes of the model legume *Medicago truncatula*. Three accessions, Jemalong 2HA, Borung and Paraggio, belong to ssp. *truncatula*, and R108 to ssp. *tricycla*. We report here that the R108 ptDNA has a ∼45-kb inversion compared with the ptDNA in ssp. *truncatula*, mediated by a short, imperfect repeat. DNA gel blot analyses of seven additional ssp. *tricycla* accessions detected only one of the two alternative genome arrangements, represented by three and four accessions each. Furthermore, we found a variable number of repeats in the essential *accD* and *ycf1* coding regions. The repeats within *accD* are recombinationally active, yielding variable-length insertions and deletions in the central part of the coding region. The length of ACCD was distinct in each of the 10 sequenced ecotypes, ranging between 650 and 796 amino acids. The repeats in the *ycf1* coding region are also recombinationally active, yielding short indels in 10 regions of the reading frames. Thus, the plastid genome variability we report here could be linked to repeat-mediated genome rearrangements. However, the rate of recombination was sufficiently low, so that no heterogeneity of ptDNA could be observed in populations maintained by single-seed descent.

## 1. Introduction

*Medicago truncatula* is a diploid model legume, a close relative of the tetraploid crop alfalfa,^1^ with its nuclear^2,3^ and plastid (AC093544) genomes sequenced and with a large collection of Tnt1 retrotransposon-tagged mutants.^4^ *Medicago truncatula* belongs to the minority of flowering plant species in which plastids are inherited from both parents.^5^ Furthermore, the *M. truncatula* plastid genome lacks the large inverted repeat (IR) encoding the plastid ribosomal RNA operon; therefore, the species belongs to the inverted repeat-lacking clade (IRLC) of the Papillionideae subfamily.^6,7^ The analyses of chloroplast genes of IR-containing and IRLC plastid genomes revealed that the synonymous substitution rate in IR genes is 2.3-fold lower than in the single-copy genes, whereas uniform substitution rates were found in genomes lacking an IR.^8^ A study of IRLC legume species revealed that the *ycf4* gene in *Lathyrus* has at least 20 times higher local point mutation rate than genes elsewhere on the plastid genome and the *ycf4-psaI-accD-rps16* region is frequently associated with a gene loss in legumes.^9^ Localized hypermutation and associated gene loss were attributed to an unusual process, such as repeated DNA breakage and repair.^9^ Short tandem and inverted repeats were also found to be a salient feature of some of the legume plastid genomes.^10^ Repeats in the intergenic region of plastid genomes are common. Interestingly, however, some legume species harbour repeats in the coding regions of *ycf1*, *ycf2*, *ycf4*, *psaA*, *psaB* and *accD* genes. Despite these repeats, the original reading frame is maintained, suggesting that the genes are functional. The repeats are species-specific, and present in only some of the species, suggesting rapid gene evolution in legumes.^9–11^

Thus far, studies of plastid genome sequences in legumes have involved only a single accession per species. The next-generation sequencing technology has enabled rapid sequencing of plastid genomes from total cellular DNA, in the absence of cloning.^12–14^ To gain insights into mechanisms operating at the species level, we used the next-generation technology to fully sequence the plastid genomes of four *M. truncatula* lines*.* Jemalong 2HA^15^ and R108-1^16^ are genetic lines with an established tissue culture system. These are ecotypes with potential for plastid transformation, a prerequisite to studying the interaction of plastid and nuclear genes and engineering the photosynthetic machinery. We have chosen cultivars Borung and Paraggio from a screen of 11 lines to be used as parental lines in a study of plastid inheritance.

We report here the finding of two alternative genome configurations in ssp. *tricycla*, represented by four accessions in a sample of eight. Furthermore, we found surprising, ecotype-specific length polymorphisms in the *accD* and *ycf1* coding regions. The alternative genome organization and intragenic length polymorphisms could be linked to the presence of short direct and inverted repeats. However, the rate of genome rearrangements is sufficiently low, so that no ptDNA heterogeneity could be observed in plants maintained by single-seed descent.

## 2. Materials and methods

### 2.1. *M. truncatula* lines

Lines Jemalong A17, A20, Borung, Caliph, Cyprus, Parabinga, Paraggio, Salernes, Sephi, DZA012, GRC020, GRC098, ESP031 and ESP098A were received from the Samuel Roberts Noble Foundation, Ardmore, OK, USA. Jemalong 2HA and R108-1 seeds were received from Pascal Ratet and Eva Kondorosi. ISV-CNRS was from Gif sur Yvette, France respectively. *Medicago truncatula* ssp*. tricycla* lines 2529 (USDA PI 660437), 2624 (USDA PI 660450), 761 (USDA PI 535614), 765 (USDA PI 535618), 1665 (USDA PI 660496), GR546 (USDA PI 516949) and W11366 (USDA PI 564941) were obtained from Stephanie L. Greene, USDA, ARS National Temperate Forage Legume Germplasm Resources Unit, Prosser, WA, USA.

### 2.2. DNA sequencing

Total cellular DNA was isolated from greenhouse-grown leaves using the CTAB method.^17^ The chloroplast genome was amplified in overlapping fragments using PCR primers modified from ref.^18^ or designed based on the reference Jemalong A17 plastid genome (GenBank AC093544) (Supplementary Table S1). Pooled PCR fragments (Supplementary Table S2) were purified on a QiaQuick MinElute kit (Qiagen, Germantown, MD, USA) and ∼8 μg of DNA was sheared in a Covaris ultrasonicator using the ‘500-bp’ programme. DNA sequence was determined on an Illumina Genome Analyzer II (Illumina, San Diego, CA, USA) using a 500-bp insert library and 80-bp paired-end reads following the manufacturer's protocol. DNA sequence of *trnQ*-*cemA* region in 10 *M. truncatula* lines (GenBank KC989947–KC989956) was determined by dideoxy sequencing of PCR amplicons.

### 2.3. Genome assembly

The plastid genomes from 80-nt paired-end reads were assembled using a combination of the Velvet v. 1.1^19^ *de novo* assembly program at hash length 71 and the Burrows–Wheeler Alignment Tool v. 0.5.9^20^ reference-based assembly programs. Missing regions between contigs were filled in by Sanger sequencing of PCR products amplified from the total genomic DNA template. Annotation was carried out using DOGMA^21^ and homologues in the *Cicer arietinum* (NC_011163), *Pisum sativum* (NC_014057), *Lotus japonicus* (NC_002694) and *Solanum lycopersicum* (NC_007898) ptDNA. Annotation of the *ycf4* gene was based on ref.^9^.

### 2.4. DNA gel blot analyses

Southern probing was carried out according to ref.^22^, except that a modified Church hybridization buffer (0.5 M Na_2_HPO_4_, 7% SDS, 10 mM EDTA, pH 7.2) was used instead of Rapid-hyb Buffer (GE Healthcare, Piscataway, NJ, USA). An amount of 1.5 μg of *Eco*RV- or *Hha*I-digested total cellular DNA was loaded per lane and probed with ^32^P-labelled Jemalong A17 PCR fragments (Supplementary Table S3).

## 3. Results

### 3.1. Sequencing of *M. truncatula* plastid genomes

**Figure 1.**
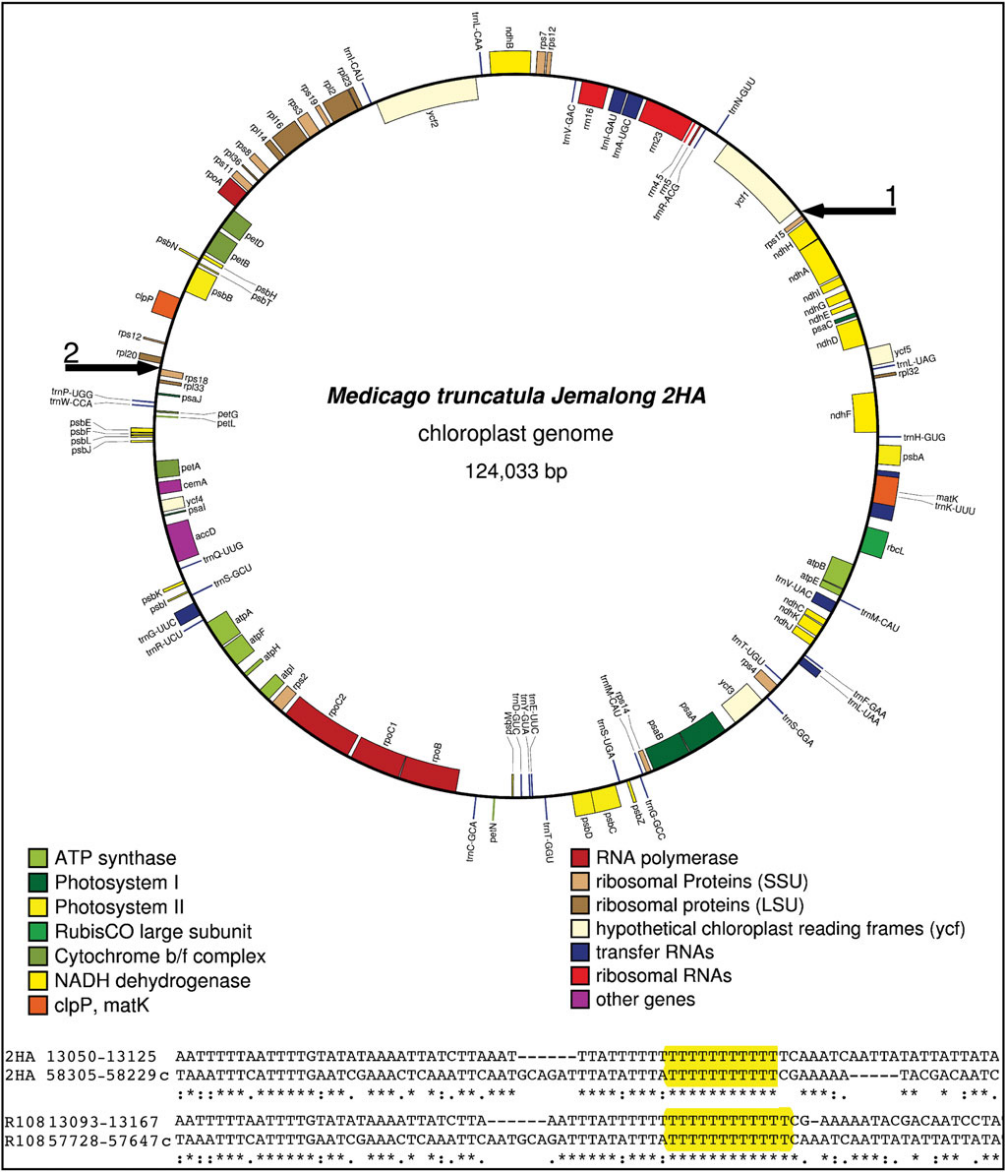
The circular plastid genome map of *M. truncatula* Jemalong 2HA line created using the OrganellarGenomeDRAW program.^23^ Genes shown on the outside of the circle are transcribed in the clockwise direction, and those shown in the inside are transcribed in the counterclockwise direction. Black arrows No. 1 and 2 outside the circle point to the inversion breakpoints in the *rps15*-*ycf1* and *rpl20-rps18* intergenic regions. Gene order in the R108 ptDNA between the arrows is in the reverse orientation. Below the map are shown the alignments of imperfect repeat sequences flanking the run of thymidine nucleotides (highlighted in yellow) containing the inversion endpoints in the R108 ptDNA and cognate sequences in 2HA.

**Figure 2.**
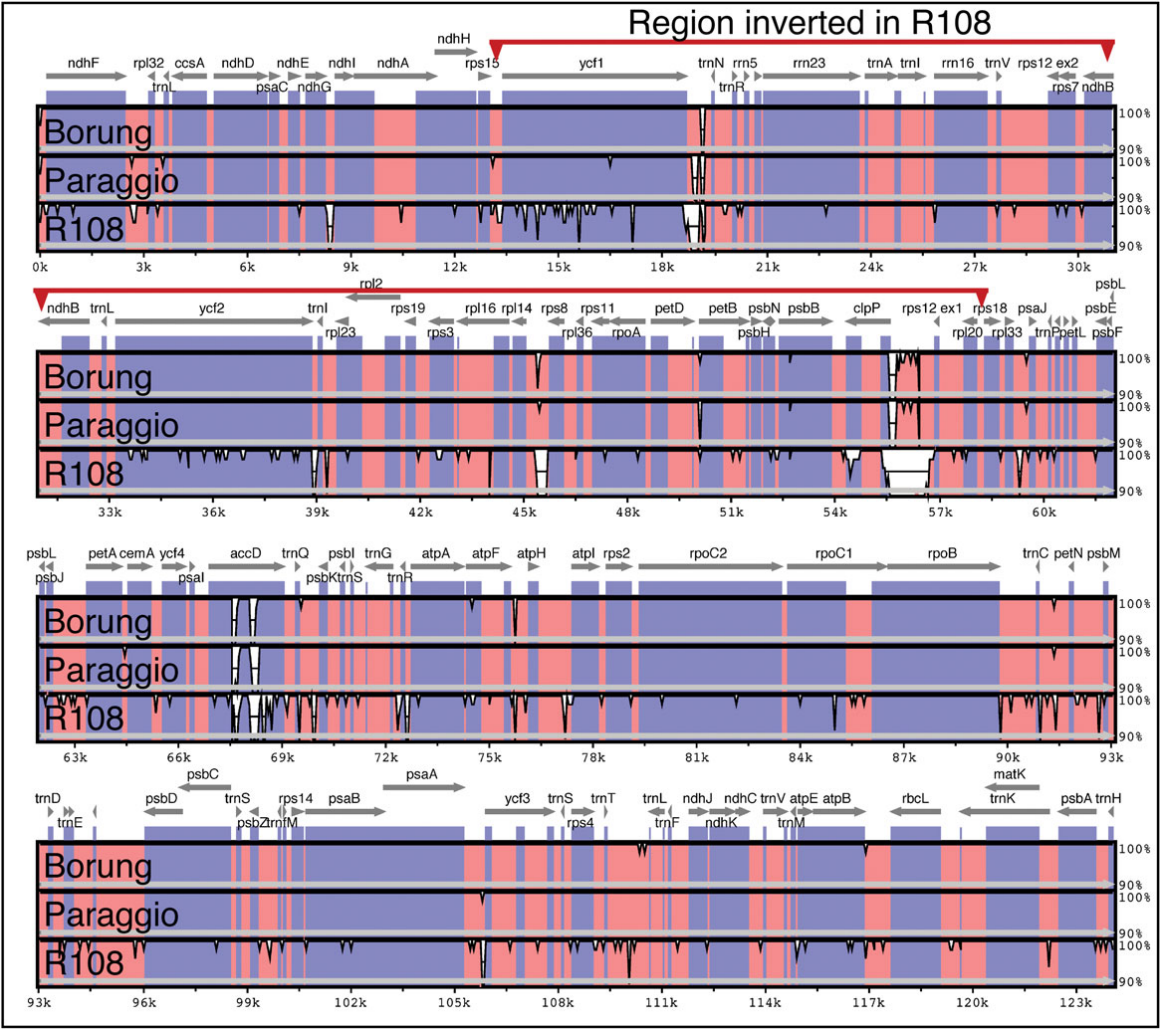
mVISTA similarity plot comparing the reference Jemalong 2HA ptDNA with the Borung, Paraggio and R108 ptDNAs. For the purpose of this figure, the R108 inversion was manually reversed. The sliding window is set to 50 bp, the consensus width to 50 bp and the consensus identity to 70%. Coding regions are in blue, and non-coding regions are in pink.

We report here the plastid genome sequence of four *M. truncatula* ecotypes: Jemalong 2HA, Borung, Paraggio and R108. We constructed paired-end libraries of PCR-amplified DNA, sequenced them on the Illumina GAII platform and assembled the plastid genome sequences from 80-nucleotide (nt) reads. The sequence ambiguities and gaps were resolved by dideoxy sequencing of PCR amplicons using total cellular DNA as template, and the genome sequences were deposited in GenBank. The three ecotypes in ssp. *truncatula*, Jemalong 2HA (124 033 bp; GenBank JX512022), Borung (123 833 bp; GenBank JX512023) and Paraggio (123 706 bp; GenBank JX512024), have the same genome organization (Figs 1 and 2). The plastid genome sequence of Jemalong 2HA (from here on referred to as 2HA) is identical to the Jemalong A17 plastid genome in the database (GenBank AC093544) other than two single nucleotide polymorphisms (SNPs) in the A17 ptDNA. Upon re-sequencing the relevant regions of the A17 ptDNA, we confirmed that the 2HA and A17 ptDNA sequences are identical. We also assembled the plastid genome sequence of the R108 line; eliminated ambiguities by Sanger sequencing of amplicons and confirmed structure by DNA gel blot analyses (Section 3.2). We report here that the R108 (ssp. *tricycla*) ptDNA (123 418 bp; GenBank KF241982) has a large ∼45-kb inversion relative to the three ssp. *truncatula* ecotypes (Supplementary Fig. S1). The inverted region is between *rps15* and *rps18*, involving all genes from *ycf1* to *rpl20.* Accordingly, the gene order at the junctions in the 2HA, Paraggio and Borung plastid genomes is *rps15-ycf1* and *rpl20-rps18*, whereas the gene order in the R108 ptDNA is *rps15-rpl20* and *ycf1-rps18*. The inversion apparently occurred via two intergenic runs of thymidine nucleotides (Ts) highlighted in yellow in Fig. 1 and Supplementary Fig. S1.

PCR amplification using the 2HA and R108 templates yielded the predicted junction fragments, confirming the genome structures shown in Fig. 1 and Supplementary Fig. S1 (primer pairs 12.909F-13.689R and S7_57758F-58.549R, and 13.689R-58647R and 12.821F-58.118F, respectively, in Supplementary Table S1). We also performed PCR amplification with non-matching primer combinations that should not have yielded specific amplicons. The 2HA template with R108-specific primers did not yield specific fragments, as expected. However, amplification of R108 template with 2HA-specifc primers (S7_57758F and 58.549R, Supplementary Table S1) yielded a specific product that, when sequenced, turned out to be a 2HA-type junction. This product apparently derived by amplification from a nuclear template that was incorporated in the nuclear genome prior to the appearance of R108 genome arrangement, confirming that the 2HA-specific genome organization is ancestral to the R108 type. In contrast, we could never amplify the R108-type ptDNA junction in the 2HA, or the other ssp. *truncatula* ecotypes.

### 3.2. DNA gel blot analyses confirm inversion in the R108 ptDNA

**Figure 3.**
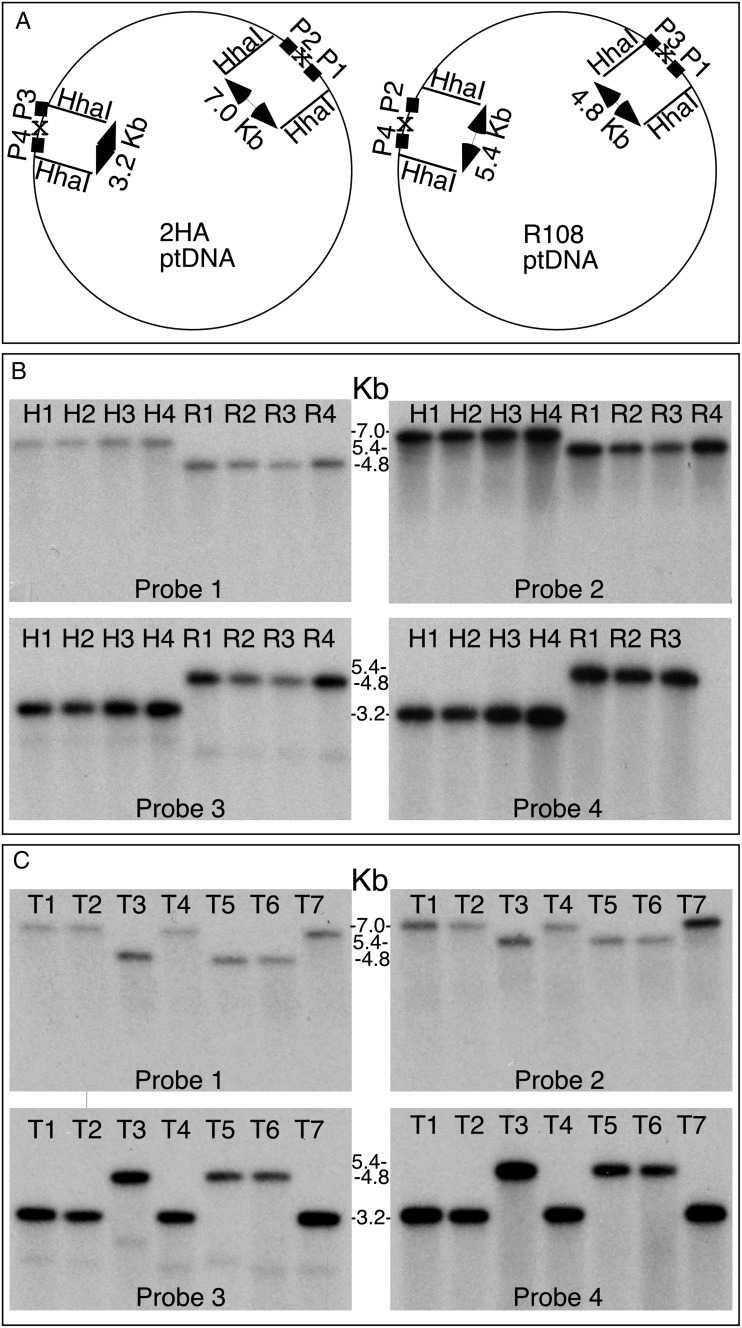
DNA gel blot analysis confirms two stable plastid genome configurations in *M. truncatula* ssp. *tricycla* ptDNA using *Hha*I polymorphic sites. (A) Schematic map of 2HA and R108 ptDNA with the position of DNA probes P1–P4. The site of inversion is marked by x. *Hha*I fragment sizes are given inside the circles. (B) Probing *Hha*I-digested total cellular DNA of four 2HA (H) and four R108 (R) plants with probes P1–P4. (C) Testing ptDNA genome structure in *M. truncatula* ssp*. tricyla* lines in *Hha*I-digested total cellular DNA using probes P1–P4. The lanes contain DNA of lines 2529, T1; 2624, T2; 761, T3; 1665, T4; GR546, T5; 765, T6; W611366, T7.

Inversion in the R108 ptDNA has been confirmed by DNA gel blot analyses. The DNA probes were derived from the regions flanking the inversion in the 2HA line (Fig. 3A). Each of the four probes could distinguish the 2HA and R108 plastid genomes when probing *Hha*I-digested total cellular DNA (Fig. 3B). Probe 1 hybridized to a 7–kb fragment in 2HA and a 4.8–kb fragment in R108. Probe 2 hybridized to a 7–kb fragment in 2HA and a 5.4–kb fragment in R108. Probe 3 recognized 3.2 and 4.8–kb fragments and Probe 4 recognized 3.2 and 5.4 kb fragments in the 2HA and R108 samples, respectively. Single fragment sizes with the probes indicate that the two genome configurations are stable, and no flip-flop recombination is taking place via the short inverted repeats. We analysed multiple individuals and obtained results consistent with only one ptDNA configuration in different 2HA and R108 plants, as shown in Fig. 3B*.* Probing of *Eco*RV digested total cellular DNA also confirmed genome structure and the absence of flip-flop recombination (Supplementary Fig. S2). The 24- and 20-nt inverted repeats in the R108 and 2HA ptDNA (Fig. 1 and Supplementary Fig. S1) are apparently too short to mediate frequent recombination that could be detected in DNA blots.

### 3.3. Survey for the inversion in ssp. *tricycla* plastid genomes

The R108 ecotype belongs to ssp. *tricycla.* To determine whether the inversion is characteristic of the subspecies, total cellular DNA was analysed from seven additional ssp. *tricycla* accessions. DNA gel blot analyses shown in Fig. 3C indicate that three of the lines (761, 765 and GR546) have an R108-type ptDNA and four others (2529, 2624, 1665 and W11366) a Jemalong 2HA-type gene order. Thus, two stable genomic isoforms are present in *M. truncatula* ssp. *tricycla*.

### 3.4. Sequencing the *accD-psaI-ycf4-cemA* region reveals variability in *accD*

Insertions and deletions in plastid genomes are typically restricted to intergenic regions. Large insertions and deletions in the *M. truncatula* ptDNAs are present in the *ycf1*-*trnN*, *rpl14*-*rps8* and *clpP*-*rps12* intergenic regions (Fig. 2). A striking feature visualized by the mVista alignment in Fig. 2 is the large number of insertions and deletions in the *accD*, and to a lesser degree in *ycf1*, coding regions.

**Figure 4.**
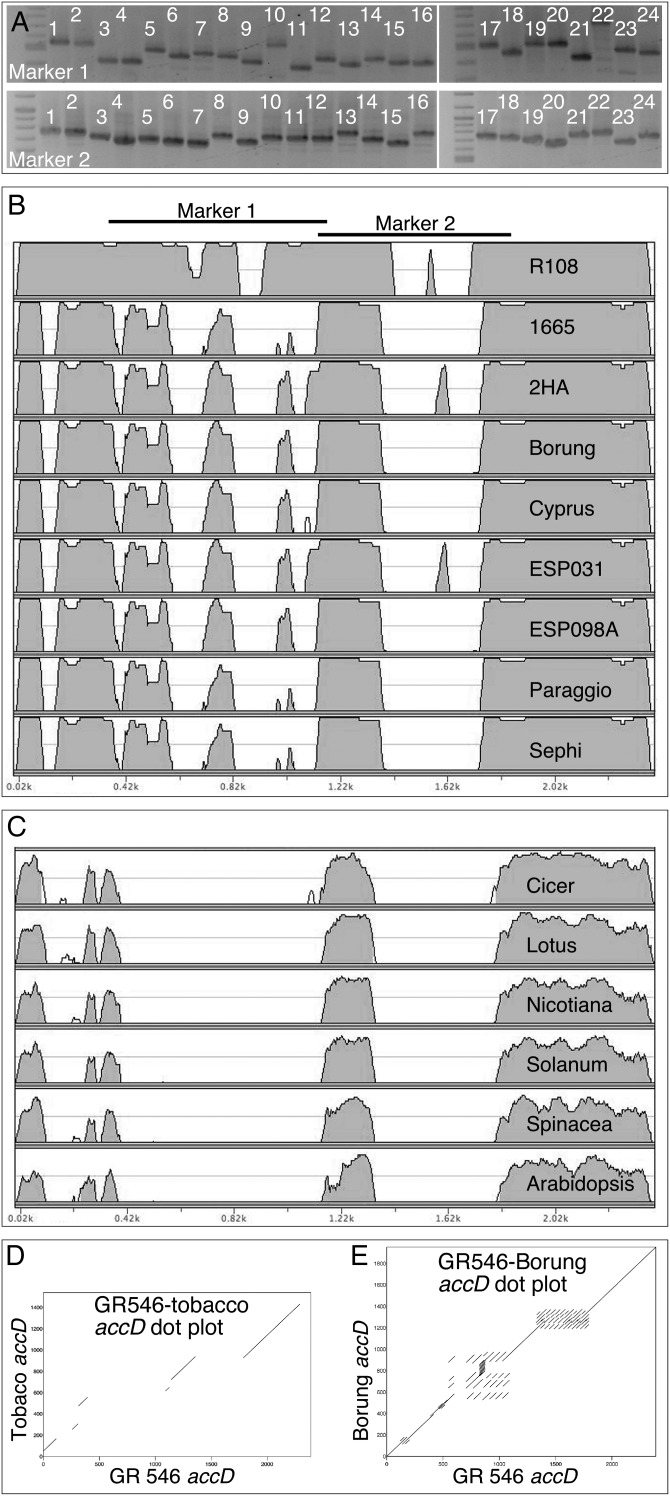
Variation in the *accD* coding region is unique to ecotypes in *M. truncatula.* (A) PCR amplicon sizes are unique to ecotypes. The lanes contain DNA from Jemalong A17, lane 1; Jemalong 2HA, 2; Jemalong A20, 3; Borung, 4; Paraggio, 5; CRE05, 6; CRE09, 7; DZA012, 8; ESP098A, 9; ESP031, 10; GRC020, 11; GRC098, 12; Caliph, 13; Salernes, 14; Sephi, 15; Cyprus, 16; R108-1, 17; 2529, 18; 2624, 19; 761, 20; 1665, 21; GR546, 22; 765, 23; W113666, 24. Marker 1 primers (5′-ATAACAACTGTCGCAGGCAACCC-3′ and 5′-TGCTTTCTGAAATCGGTATTGATAGTTCC-3′) amplify the region 67980–68764 and marker 2 primers (5′-GTGCCTGTTTGAACCGCATCCAG-3′ and 5′-TTTCGCATTTGTGGGTTGCCTGC-3′) amplify the region between 67468 and 68014 in the Jemalong 2HA genome. (B) The mVISTA similarity plot of *accD* coding regions compared with the longest reading frame in GR546. The window is 50 bp, the consensus width is 50 bp and the consensus identity is 70%. (C) The mVISTA similarity plot of *C. arietinum* (NC_011163), *L. japonicus* (NC_002694), *N. tabacum* (NC_001879), *S. lyco-persicum* (NC_007898), *Spinacea oleracea* (NC_002282) and *Arabidopsis thaliana* (NC_000932) compared with the longest *accD* reading frame of *M. truncatula* GR546 accession. (D) Dot matrix plot comparing the *accD* coding region of *N. tabacum* and GR546, and (E) GR546 and Borung to visualize repetitive DNA using the criterion of 27 matching bases per 30 bp window.

**Figure 5.**
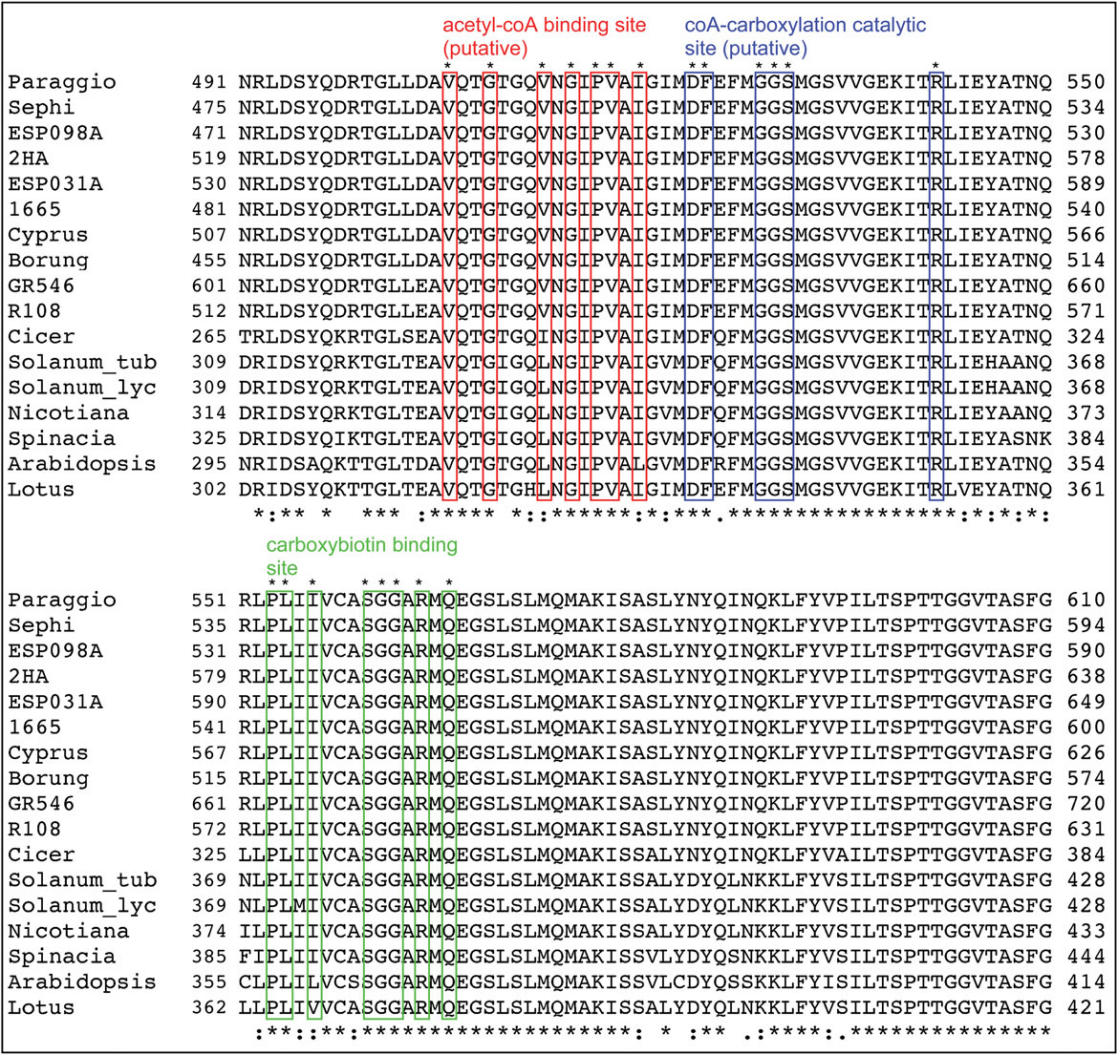
Conserved amino acid sequence motifs of accDs. Shown is the ClustalW alignment of the region containing the putative acetyl-CoA binding site, the CoA-carboxylation catalytic site and the carboxybiotin-binding site^24^ in the *M. truncatula* accessions and other flowering plant species. For the *Solanum tuberosum accD* gene sequence, see GenBank AF069288; the rest of the GenBank accessions are given in the caption of Fig. 4. This figure appears in colour in the online version of *DNA Research*.

Intrigued by the insertions and deletions in the *accD* coding regions, we developed PCR markers spanning two variable regions and surveyed 24 *M. truncatula* ecotypes. We found that most, if not all, ecotypes could be distinguished by the combination of the two markers (Fig. 4A). To gain further insights into *accD* coding region variability, we sequenced the *accD-psaI-ycf4-cemA* region in 10 *M. truncatula* ecotypes (GenBank KC989947–KC989956). Alignment of the 10 *accD* coding regions revealed extensive length variation: ecotype GR546 had the longest (2391 bp, KC989955) and Borung the shortest (1953 bp, KC989949) *accD*, encoding 796 and 650 amino acids, respectively. Alignment of *M. truncatula*, other legume and angiosperm *accD* coding regions revealed islands of sequence conservation, including sequences at the *N*- and *C*-termini (Fig. 4B). Species-specific repetitive DNA has been reported in the *P. sativum* and *Lathyrus sativus accD* coding regions.^9^ Therefore, we used dot matrix plots to visualize repetitive DNA in the *accD* coding region of *M. truncatula* ecotypes (Fig. 4E). We have found that the variable regions contain a large number of complex repeats that are unique to the ecotype. The tobacco (512 amino acids), *Arabidopsis* (488 amino acids) and other angiosperm *accD* genes are significantly shorter (Fig. 4C) and lack repeats (Fig. 4D), suggesting that the variable protein regions encoded in the DNA repeats are not important for gene function. Interestingly, the reading frame in the *accD* genes has been conserved, suggesting that the genes are functional. In the potato plastid *accD*, three functionally relevant sites were identified: a putative acetyl-CoA binding site, a CoA-carboxylation catalytic site and a carboxybiotin-binding site.^24^ Each of the sites is clustered at the C-terminus of the protein, and they are conserved in all *M. truncatula* accessions (Fig. 5).

Comparative analyses of six different legume plastid genomes revealed extensive variation in the *psaI-ycf4-cemA* region, including length variation in *ycf4* and/or the loss of *psaI*, *ycf4* or *cemA* genes.^9^ We did not find variation in the gene content in the 10 sequenced *M. truncatula* ecotypes (GenBank KC989947–KC989956).

### 3.5. Length variation in the *ycf1* coding region

We aligned the *ycf1* coding region in our four sequenced *M. truncatula* ptDNAs and screened them for indels. We have found that each of the coding regions were unique to the ecotype (Supplementary Fig. S3). The relatively large (5.3-kb) gene contains 10 polymorphic regions in the four lines, some of which are flanked by repeats, as in the *accD* gene. However, the repeats are less complex than in the *accD* gene, and the indels are much shorter. Unlike the length of *accD*, the overall length of *ycf1* coding region is conserved in angiosperms (Supplementary Fig. S3).

## 4. Discussion

### 4.1. Two stable plastid genome configurations in *M. truncatula*

Sequencing of multiple plastid genomes in rice (*Oryza*)^25^ and *Jacobaea vulgaris*^26^ revealed that intraspecies genome variability is typically restricted to SNPs and microsatellites in intergenic regions, and silent point mutations within coding regions. Particularly well conserved are plastid genomes in the Solanaceae family where the ptDNA of two sequenced cultivars in tomato (cv. IPA6 and cv. Ailsa Craig)^27^ and tobacco (cv. Bright Yellow and cv. Petit Havana)^28^ are identical to the nucleotide and the ptDNA of the allotetraploid *Nicotiana tabacum* and its maternal progenitor, *Nicotiana sylvestris*, differ only by seven sites.^29^ In contrast, our probing of plastid genome structure in *M. truncatula* revealed two stable plastid genome configurations. The 45-kb inversion is through a run of Ts nested in a short (20–24 nt) imperfect repeat (Fig. 1 and Supplementary Fig. S1). Finding two alternative genome configurations in a species, aside from an early report in pea,^30^ to our knowledge, is unprecedented. The plastid gene order in the IR-containing legume *L. japonicus*^31^ and the closely related IRLC legume *C. arietinum* (chickpea)^32^ is the same as in the three ssp. *truncatula* accessions. Therefore, the R108 ptDNA genome organization is derived, generated by an inversion via the short direct repeats (Supplementary Fig. S1). Compared with the ancestral gene order, reorganization of the ptDNA in another legume, *Trifolium subterraneum*, is much more extensive, involving 14–18 inversions of 16 gene clusters. The endpoints of rearranged gene clusters are flanked by repeated sequences, as in *M. truncatula*, or tRNAs and pseudogenes.^33^ The ptDNA of the legume species *P. sativum* and *L. sativus* contain five and three inversions, respectively.^9^ In these legume species, the ptDNA of only a single accession has been studied. We predict, based on our finding of two stable plastid genomes in *M. truncatula*, that a survey of multiple accessions in these legume species is likely to uncover multiple genome configurations.

### 4.2. Intraspecies variation in the *accD* and *ycf1* coding regions

The plastid-localized *accD* genes encode the β-carboxyl transferase subunit of acetyl-CoA carboxylase (ACCase). It is an essential gene in tobacco, in which attempts at deleting the gene failed to yield stable knockout plants.^34^ Interestingly, *accD* has been lost independently at least six times from the plastid ge-nome of angiosperms, concurrent with the evolution of a nuclear copy.^35^ Well characterized is loss of the *accD* gene from the Gramineae plastid genome, where the prokaryotic type (heteromeric) plastid ACCase was replaced with a eukaryotic-type homomeric form in the nucleus.^36,37^ The evolutionary loss of *accD* gene from the plastid genome of *Trifolium repens*^9^ and *Trachelium caeruleum*^35^ was also concurrent with the transfer of an *accD* copy to the nucleus. In *T. repens*, the nuclear copy of *accD* is fused with the plastid lipoamide dehydrogenase; in *T. caeruleum*, a truncated carboxylase domain (331 amino acids), containing only ∼250 conserved amino acids, is fused with a transit peptide. The *accD* genes in the sequenced *M. truncatula* ptDNA are larger, ranging in size from 650 to 796 amino acids (Fig. 4B), and always include the conserved carboxylase domain. Each of the 24 *M. truncatula* ecotypes in our survey appears to have a unique *accD* gene (Fig. 4A). However, the reading frame in each of the 10 sequenced lines has been maintained (Fig. 4B). The variable domains constitute the polymorphic regions containing a cluster of complex repeats (Fig. 4E). The compatibility of length variation with *accD* function explains why so many alleles are present in the different ecotypes. Intragenic expansion and contraction of the *accD* coding region appear to be linked to the presence of repeats. Frequent length polymorphism is likely to be generated by replication slippage, as described in the repeat-containing *Oenothera* ptDNA. Unlike in *M. truncatula*, the repeats in the *Oenothera* ptDNA are found in intergenic regions.^38,39^

The *ycf1* gene is also essential in tobacco, as no stable transplastomic plants lacking the *ycf1* gene could be obtained.^40^ The *ycf1* gene encoding a 214–kDa protein of the Tic complex^41^ is also tolerant to insertions and deletions, but the size of insertions and deletions is much smaller than in the *accD* gene, 2–15 amino acids in ∼10 polymorphic regions. The reading frame is always maintained, suggesting that the genes are functional. The repeat structure in the *ycf1* coding region is less complex than in the *accD* gene, typically a pair of short (5–8 nt) tandem repeats flanking the variable region.

### 4.3. Next-generation sequencing of plastid genomes

We assembled the plastid genome sequence from 80-nt reads of PCR-amplified DNA. PCR amplificons of the ptDNA could be readily obtained for the ssp. *truncatula* genomes, which have the same general organization as the published A17 ptDNA. Inversion in the R108 ptDNA was suspected based on the absence of large PCR amplicons from the regions containing the inversion using A17 primers (note the absence of fragments with primers 9.7F/15.5R and 49F/50.8R in Supplementary Table S2 in R108 line). However, ancestral ptDNA copies in the nucleus were a complicating factor in the R108 line, because we obtained small PCR fragments for both configurations at the *rpl20-rps18* junction. The controversy could ultimately be resolved by DNA gel blot analyses detecting only high-copy ptDNA, confirming the inversion in the R108 plastid genome. The presence of a few copies of ptDNA fragments covering the entire genome in the nucleus is well documented in many species, including tobacco,^42^ *Arabidopsis*^43^ and maize.^44^ Best characterized is the nuclear plastid DNA (NUPTs) in the rice nucleus, where sequential transfer of ancestral ptDNA could be shown.^45^

### 4.4. Utility of genome sequence for genetic analyses and biotechnology

The ptDNA sequence information we report here provides new markers to study plastid inheritance,^5,46^ and for the design of plastid transformation vectors where homology between the vector targeting sequences and recipient ptDNA is important for efficient incorporation of the transforming DNA.^47,48^ Our study of complete plastid genomes of multiple accessions in *M. truncatula* revealed a significant intraspecies ptDNA variation. Therefore, it will be particularly important to obtain subspecies-level ptDNA sequence information for vector design in clades, which have highly rearranged plastid genomes, such as the Geraniaceae,^49,50^ Campanulaceae,^51–53^ Oleaceae^54^ and Fabaceae.^33,55^

## Supplementary data

Supplementary data are available at www.dnaresearch.oxfordjournals.org.

## Funding

This work was supported by the United States Department of Agriculture, National Institute of Food and Agriculture, Biotechnology Risk Assessment Research Grant Program Award 2010-2716 to P.M. C.G. was supported by the Waksman Institute of Microbiology Busch Predoctoral Fellowship and a teaching assistantship from the Division of Life Sciences, Rutgers University.

## Supplementary Material

Supplementary Data
