# Supplementary material for: Two Distinct Plastid Genome Configurations and Unprecedented Intraspecies Length Variation in the accD Coding Region in Medicago truncatula
Source: DNA Res. 2014 Mar 17;21(4):417–27. doi: 10.1093/dnares/dsu007 (PMC4131835; doi:10.1093/dnares/dsu007)
Supplement: Supplementary Data [file supp_21_4_417__index.html]

Two Distinct Plastid Genome Configurations and Unprecedented Intraspecies Length Variation in the accD Coding Region in Medicago truncatula — Two Distinct Plastid Genome Configurations and Unprecedented Intraspecies Length Variation in the accD Coding Region in Medicago truncatula — Supplementary Data 

# Two Distinct Plastid Genome Configurations and Unprecedented Intraspecies Length Variation in the *accD* Coding Region in *Medicago truncatula*

## Supplementary Data

Supplementary Data

**Files in this Data Supplement:**

- Supplementary Figure 1 - pdf file
- Supplementary Figure 2 - pdf file
- Supplementary Figure 3 - pdf file
- Supplementary Table 1 - pdf file
- Supplementary Table 2 - pdf file
- Supplementary Table 3 - pdf file
- Supplementary Data - Doc file
