# Supplementary material for: Two Distinct Plastid Genome Configurations and Unprecedented Intraspecies Length Variation in the accD Coding Region in Medicago truncatula
Source: DNA Res. 2014 Mar 17;21(4):417–27. doi: 10.1093/dnares/dsu007 (PMC4131835; doi:10.1093/dnares/dsu007)
Supplement: Supplementary Data [file supp_dsu007_dsu007supp_fig2.pdf]

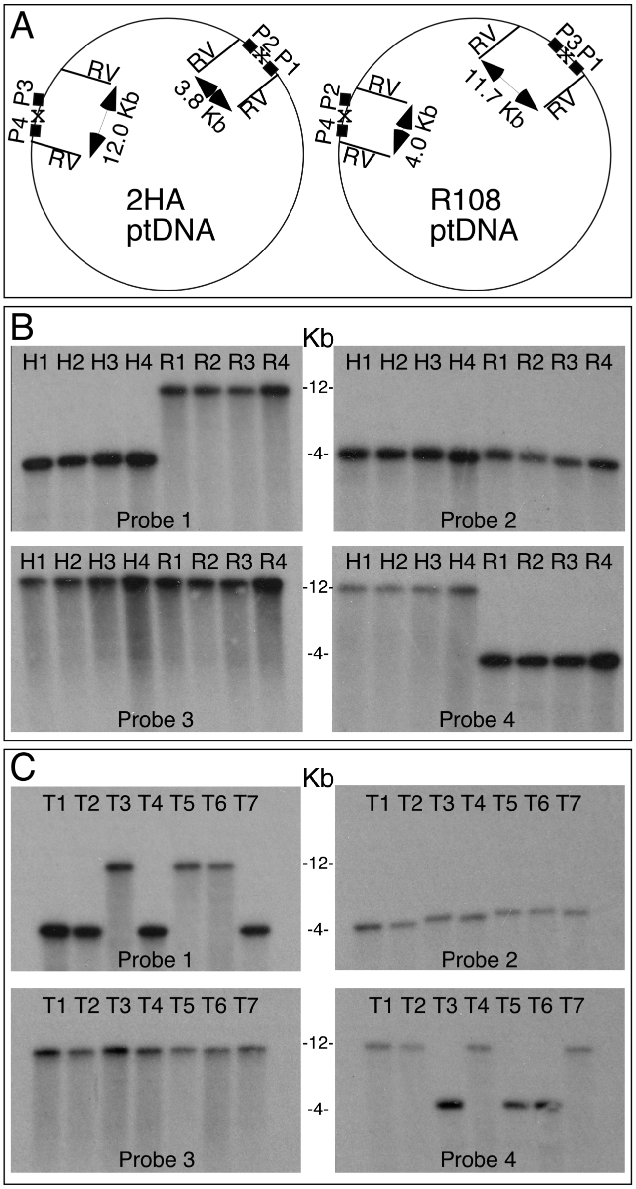


**Supplementary Figure S2.** DNA gel blot analysis confirms two stable plastid genome configurations in *M. truncatula* ssp. *tricycla* ptDNA using *Eco*RV polymorphic sites. (A) Schematic map of 2HA and R108 ptDNA with the position of DNA probes P1-P4. Site of inversion is marked by x. *Eco*RV fragment sizes are given inside circles. (B) Probing *Eco*RV -digested total cellular DNA of four 2HA (H) and four R108 (R) plants with probes P1-P4. (C) Testing ptDNA genome structure in *M. truncatula* ssp. *tricyla* lines in *Eco*RV -digested total cellular DNA using probes P1-P4. The lanes contain DNA of: line 2529, T1; 2624, T2; 761, T3; 1665, T4; GR546, T5; 765, T6; W611366, T7.
