# Supplementary material for: Two Distinct Plastid Genome Configurations and Unprecedented Intraspecies Length Variation in the accD Coding Region in Medicago truncatula
Source: DNA Res. 2014 Mar 17;21(4):417–27. doi: 10.1093/dnares/dsu007 (PMC4131835; doi:10.1093/dnares/dsu007)
Supplement: Supplementary Data [file supp_dsu007_dsu007supp_fig3.pdf]

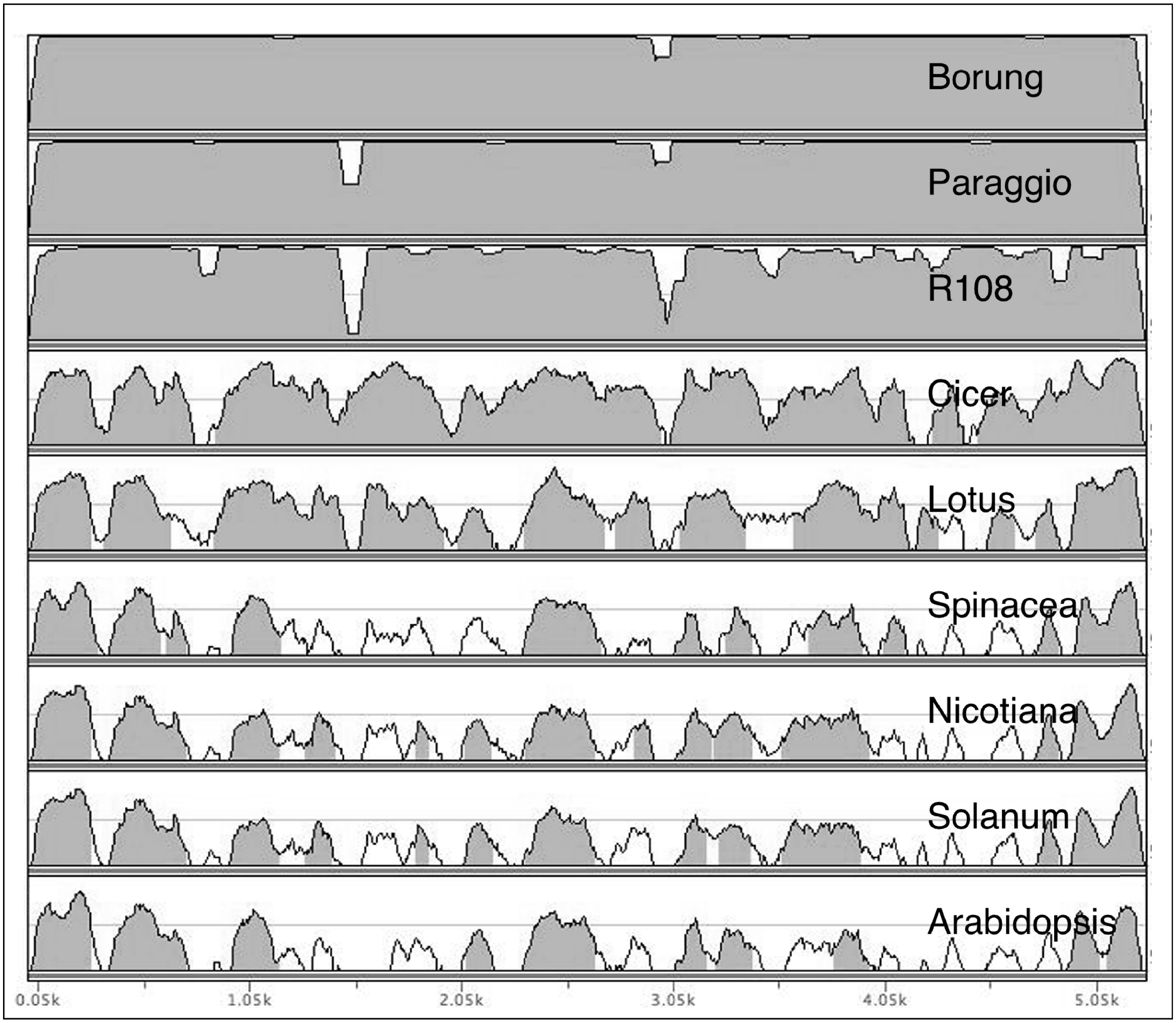

Supplementary Figure S3. The *ycf1* mVISTA similarity plot of Borung, Paraggio, R108 and angiosperm species *Cicer arietinum* (NC_011163), *Lotus japonicus* (NC_002694), *Nicotiana tabacum* (NC_001879), *Solanum lycopersicum* (NC_007898), *Spinacea oleracea* (NC_002282) and *Arabidopsis thaliana* (NC_000932) compared to the *M. truncatula* 2HA line. The window is 100 bp, the consensus width is 100 bp and the consensus identity is 70%.
