# Supplementary material for: Two Distinct Plastid Genome Configurations and Unprecedented Intraspecies Length Variation in the accD Coding Region in Medicago truncatula
Source: DNA Res. 2014 Mar 17;21(4):417–27. doi: 10.1093/dnares/dsu007 (PMC4131835; doi:10.1093/dnares/dsu007)
Supplement: Supplementary Data [file supp_dsu007_dsu007supp_table1.pdf]

Table S1. Primers to amplify the *M. truncatula* plastid genome fragments

Primer start	Primer name	Primer sequence
718	M16A_718F	TCCTATGGACCCGACGAAGAAAG
731	0.731R	TCGGGTCCATAGGAATTTCTTTCAGCC
1123	M16A_1123R	AGCTTGTTCCACAGCAGGATTAAC
1130	1R	CAATAATAGCTTGTTCCACAGCAG
1235	1.235F	GCGGTTTTTGTAATTGGGGCATGTTTTG
1258	1.2R	ACATGCCCCAATTACAAAAACCGCT
1717	1.717F	TCCGCAAAATAACAAGAGGGCGCAC
1905	1.90-1F	CATTTGCCGCAATAGGACGTGTGAAC
1906	1.90F	ATTTGCCGCAATAGGACGTGTGAACC
1910	1.9F	GCCGCAATAGGACGTGTGAACCA
2056	2.056F	TCTCAAATAGCCTTGATCATGAGAC
2257	M5_2257F	TTGTTCTGAATCTTTGCACAATCGTTCC
2293	M5A_2293F	ACTAGGAAAAGCCCACATACGGCG
2296	M4_2296F	AGGAAAAGCCCACATACGGCGAAT
2296	M4B_2296F	AGGAAAAGCCCACATACGGCCAAT
2585	M4A_2585R	TGGAACGATTGTGCAAAGATTCAGAACA
2585	M4B_2585R	TGGAACGATTGTGCAAAGATTGAGAACA
2616	M4_2616R	TGGCCGATTTTGACTTTGTACTTTCCA
3098	3.098R	TCGATGTGAAAGACATTTGTTCTG
3204	M6_3204F	TTGGACAGCATTGAAAGCCTTTTCATTAG
3223	M5_3223R	AGGCTTTCAATGCTGTCCAATATCCC
3228	3.228R	TGAAAAGGCTTTCAATGCTGTCC
3617	3.617F	GCCGCTATGGTGAAATTGGTAGACACGC
3680	M6_3680R	CGAACCGAGATGCTCTAGCACTGC
3686	3.6R	CGGACTCGAACCGAGATGCTCT
3692	M5A_3692R	GCTACTCGGACTCGAACCGAGATGC
3696	3.696R	TGCCGCTACTCGGACTCGAACC
3696	3.696R	TGCCGCTACTCGGACTCGAACCG
4262	4F	CTATGCAGCTCTTTTATGtGGATC
4562	4.562F	ATGGGCTAACGAGGCATGGGGGTC
4564	4.4F	GGGCTAACGAGGCATGGGGG
4566	4.5F	GCTAACGAGGCATGGGGGTCCT
5127	5R	CCTTATACCCGTAATAGGTATTG
5561	5.561R	AGTGATACTGGGCTCAACGGGGCC
5805	M17_5805F	ATGTGCCTCTCCGTGGGTATCCG
6236	M17_6236R	TTAGCCACTTTAGCGGCTCAGCC
6497	6.4R	GTTTTCCCAATAGCCGCAGGTTCCT
6721	6.721F	TCGGACAAGCGGACTCACACCTC
7286	7.286R	GCTATCGGACTGGCTATTGTTTCCGC
7418	7.418F	GGGCTCTAACCATATTTCGGCTCGTG
8229	8.229R	TGGATTTGCCTGAAACGCTACACG
8576	8_576F	TGACATTGGTAAACGGCCCAAAGC
8875	8.875R	ACATCGGAACGTTTCCGCGGT
9555	9.555F	AGGTTGACGCCACAAATTCCACCC
9555	9.555F	AGGTTGACGCCACAAATTCCACCCC
9562	9.562F	CGCCACAAATTCCACCCCCAAAAACC

9580	9.580R	GGGGGTGGAATTTGTGGCGTCAACC
9597	9F	TTGCGCATCAACTATATCAACTGTAC
9702	9.7 F	CTCATACGGCTCCTGGAAAG
10523	10.523F	CTCCAATCTTGGGTTAAATGTCCTCTTCTC
10890	10R	GTGTCTTATCAATATCTCTACGTGC
10918	10.918F	TCGATTGAGCAGCAGCTCGTAGACC
10919	S1_10919F1	CGATTGAGCAGCAGCTCGTAGACC
10922	10.922F	TTGAGCAGCAGCTCGTAGACCACCC
10929	10_929F	GCAGCTCGTAGACCACCCAAAAAGG
10943	10.94R	TGGTCTACGAGCTGCTGCTCAA
10946	10.9R	GGGTGGTCTACGAGCTGCTGC
10948	10_948R	TTGGGTGGTCTACGAGCTGCTGCT
11230	M7_11230F	CGGGTCCAATACGCTGTTGTATCCC
11532	11.532F	ACCAGGTGGACGAATTTTCCATCTCC
11558	M7_11558R	TGGAGATGGAAAATTCGTCCACCTGG
11596	11_596F	AATTCCCCCTTGGGGGCCTCAAC
11750	11.750F	GCCCACCTGGAATTCCTTCCAAAGC
11852	11.852F	GCCATTGAACTTCCCAATCAAATTCCTC
11918	S1_11918F2	GTATTCCGGAAGCCCGAAGCATCG
11937	11.937R	GCTTCGGGCTTCCGGAATACAATGG
11994	11_994R	AGCCGTAGGTGTTGTTGGTAGAGAG
12217	12.127F	GGGGTTTGTGCACCAATATCTGCC
12336	S1_12336F3	TCCCAATTGTTCCGGTCCATTTACGG
12369	12.369R	AGCAATAACCGTAAATGGACCGGAAC
12706	12.706F	AACGAATTTGTGAATCCCGAATACCC
12708	12.708F	CGAATTTGTGAATCCCGAATACCCAAC
12734	12.734R	GTTGGGTATTCGGGATTCACAAATTCG
12800	12.800R	ACGGCAACGACTACTGTCTTATTTGTC
12821	12.821F	ACCCCTTTGAGATAAATAGTCTTTTGGGTG
12909	12.909F	TCAACTGATCCGGGGTTTTCTTCTTC
12932	12.932R	AGAAGAAAACCCCGGATCAGTTGAA
13461	13.461R	TGGTTTAATACCCATAATGGAAGCCATTTC
13666	13_666F	AGGGACACTGAGGACTTGAAGGAAAGGC
13777	13_777F	CCAACTCCAACTTTTTCGCGTCTTACTC
13803	13_803R	AGTAAGACGCGAAAAAGTTGGAGTTGG
13804	13_804R	GAGTAAGACGCGAAAAAGTTGGAGTTGG
13921	S1_13921R	GGACACTGAGGACTTGAAGGAAAGGC
13962	14 F	CCAGCATTTCTATATCTAGCTCT
14512	14R	GATTTCTTATAATTACAACATCTCTATCC
14743	14.743F	AGCCACACGATGTTCATTGATTCTATTCC
15570	15.570F	TCGATTCCATTTTAACGGGTGATTCCTC
15590	15.5R	CACCCGTTAAAATGGAATCG
15883	15_883R	TGAGGGTTTCCCAAGAGACCCACT
16086	16_086F	CGGATACTGGAATGCCCTGATAGGTGC
16574	16_574R	AGGGATCTATGCGCCCTCTAAGGC
16574	16.574R	AGGGATCTATGCGCCCTCTAAGGC
16806	16.806F	ACCATCGAGGAACTTGTTTACTGATTTCG
16900	16_900R	CGAGGCAATTAGAACTGATCAGAACAACC
17269	S1_17269R2	TCCTTTCTTGAATGGACCTTTTCGTGG

18382	18.38-1F	ATACGGTAGAGTTAGGACAGTGATTGT
18383	18 F	TACGGTAGAGTTAGGACAGTGATTG
18384	18.38F	ACGGTAGAGTTAGGACAGTGATTGTATG
18418	S2_18418F	AATCAAAGCCAAATGCAAAGGCGC
18493	18.493F	TGCTGCTATTTCCGTCTCGGTCCC
18532	18_532R	CCGGGTTATGGACAAAGGGACCGAG
19340	S2_19340R	GCCTCATTTCATAGGTAAATTCAAAGTGGC
19442	19.44-1R	CCGCTCTACCACTGAGCTACTGAGGA
19444	19.44R	GACCGCTCTACCACTGAGCTACTGAG
19449	19_449R	CAGCCGACCGCTCTACCACTGAG
19450	19R	ACAGCCGACCGCTCTACCAC
19584	M20_19584F	ACGGGTTACCCCTGTCATTGTTTC
19651	M29_19651F	TGGAGACTCGCGGTCAATACC
19980	M20_19980R	GTGGAAGCAAAGCTATGGAACTTAGC
20121	20.121R	AGACTGGGTGGGGCCTGTAGCTC
20574	M29_20574R	GGCAGAGGCCTTTGGTGTCC
21754	22F	CGCTACCTTAGGACCGTTATAGTTAC
22969	23R	GAAACTAAGTGGAGGTCCGAACCGAC
24524	M30_24524F	TCTGACCAACCGCCCATCCTAC
24828	24.828F	TGGACTTGAACCAGAGACCTCGCCC
25330	25F	AtAGAAAGTTGGATCTACATTGGATC
25472	M8_25472F	CAGGCGCCGATGAGCACATTGAAC
25537	M30_25537R	GTCGTTGTGCCTGGACTGTGAG
25538	M8A_25538F	GCAATTATCAGGGGCGCGCTCTACC
25926	M8_25926R	GGTAGCCGTACTGGAAGGTGCGAC
25938	25.938F	TCACTCCAGTCACTAGCCCTGC
25949	25.949F	ACTAGCCCTGCCTTCGGCATC
25951	25.951F	TAGCCCTGCCTTCGGCATCC
25972	M8A_25972R	GGGGATGCCGAAGGCAGGGCTAG
25987	25.987F	GGTAACGACTTCGGGCATGGCC
26687	26R	CGACACTGACACTGAGAGACGAAAGC
27211	M31_27221F1	TAGCAGCCGTTTCCAGCTGTTG
27700	27.70F	TCGAACCGATGACTTCCACCACG
27723	27.723R	ACGTGGTGGAAGTCATCGGTTCG
27751	M28_27.751F	ACCCCTTCCCTTGCCCCTATC
28115	M31_28115R	TCGCAAATGGTAAGATCGTTGGC
29090	M28_29.090R	TCACCTCATACGGCTCCTCG
29191	29.2F	TGCCAGAGTACGCTTAACCTCTGG
29223	29F	TCACTGCTTATATACCCGGTATTGGC
29367	29.367R	TGGCGATCCTTTACTCCGACAGC
29465	29.465R	CATCGCCACTCCCTTTGGCAG
29863	29.863R	TCGCCACTCCCTTTGGCAGCATCC
29880	29.880R	GCTTTTTGCGTATGGCATCGCC
29922	30R	GTGCAAAAGCTCTATTTGCCTCTGCC
33211	33F	GGAACAGATAGTAACAACCATCTCC
33224	33.22F	ACAACCATCTCCTCCGGGAAAAGCCA
33227	33.2F	ACCATCTCCTCCGGGAAAAGCC
34086	34R	GATCTAGTTCATGGCcTATTAGAAGT
34177	34.16R	CGGATCCGTCGCTCAAAACCTTTGG

34182	34.1R	TCTGCCGGATCCGTCGCTCA
34189	34.18-1R	GAGTTGTTCTGCCGGATCCGTCG
36266	M22_36266F	CTAATATCATGAAAAATGGATACAAATTCT
36395	M22_36395R	AATTGTTACTTACCCGGACAGG
37651	37F	ATGTTGGTTGATCGTGTATTTCCTTG
38345	38.3R	ATCCTACAAGAGCCGGCCGGT
38444	38R	GATACAGGAGCGAAACAATCAACtta
38654	S3_38654F	CGATTGCCTTTGCGTGAAAGTACG
39083	S3_39083R	GCCATGGATGCTTCGTGGGAATCC
39095	M1A_39095F	AGCGCCCAACTCATAATTGGCG
39143	M1B_39143F	TGCACGCCAATAGAACCCTACCTCC
39155	M1_39155F	GAACCCTACCTCCCATAAAGTC
39469	M1A_39469R	TGACAGATGGTCAGAACTTCGTCTCG
39472	M1B_39472R	CTCTGACAGATGGTCAGAACTTCGTCTC
39503	M1_39503R	CCATTGATACAGAGCCAATCGG
41324	41F	TGGGGTTATCCTGCACTTGGAAG
42332	42R	AGTCTGAAACCAAGCGGATGTATT
45005	45F	TCACTAAAATAGTTTCATTAGCACCCG
45005	S4_45005F	TCACTAAAATAGTTTCATTAGCACCCGAGG
45790	S4_45790R	TTTTCGATTCATGTCAGCATTGCGTATC
46111	46R	TTCGCCGCCGATTCTTTCTAATC
49091	49F	ATTATGACCCATCCCTTTAGCC
49095	49.09F	TGACCCATCCCTTTAGCCAATTTCGC
49892	S5-49892F	TTCGGAGAGTTCCATTCAACTATTCGTG
50131	51R	GCACTTTCCAATGATACGTAAGC
50131	50.13R	GCACTTTCCAATGATACGTAAGCAAGGC
50220	S5_50220R	AGCGCTAGTGTGGGACAATCTACC
52000	52.000F	CTGCTTTTGGGCAACCCTCTCAAC
53061	53F	AGTATCCCTGATCCCATTGATAACG
53252	53_252F	TGGAAAAGGCCCGCTAATATGCCC
53761	53.761R	TCCCTCTGACCCTGTTCTTGACCC
53891	53R	TGCCTTGGTATCGTGTTCATAC
53969	M9_53969F	GGACCTTCCCCTTTTAGTAAATTATCTCGC
54309	M9_54309R	CTTTGGAGTGAGAACGTAAGGAGACAG
54343	54_343F	ATGCCAATTGGTGTTCCAAAAGTACCC
54796	M3_54796F	CGAGTTCGATATGCAAAACTTATTATTTTTC
54914	M3_54914R	GTGACACTAAAATAGAGTATTTTATTTATATAA
54921	S6_54921F	TTTTTGCTTTCGTTTTTCATAGCAGAGAC
55438	55.438F	TGCACAAAGAACAGGCAAAGCTTC
55505	55.505F	GCAGAAGAAGCTGAAGCCCACGG
55505	55_505F	GCAGAAGAAGCTGAAGCCCACGGA
55528	55.528R	TCCGTGGGCTTCAGCTTCTTCTGC
55937	55.937R	GAACGTATGTTGGAATGATAACATAAC
55964	55.964F	CGGAATCGTATGAATAAACAATTTGTTAG
56346	56.346R	CCATTATCATTATATGAATGGTAGAATAC
56890	57F	ATTAGAAACACAAGACAGCCAAT
56973	S6_56973R	AGTACATGTTCCTCGGCGCTGAG
57758	S7_57758F	TACATCAAGCTTTCGCGGAGCTC
57759	57.759F	ACATCAAGCTTTCGCGGAGCTCATTC

57759	57.7F	ACATCAAGCTTTCGCGGAGCTCA
57821	57.8F	AGCTTTGGTTTCGGCTCATCGGG
57821	57_821F	AGCTTTGGTTTCGGCTCATCGGG
57844	57_844R	TCCCGATGAGCCGAAACCAAAGC
58046	58.046R	CTCCTTGGCAATCATAAAAAGACAATTCCC
58118	58.118F	TAAAATAGAATTCATATGATAAAGTTATC
58202	58.202F	TTTCTTACAACGCAACATCCTCAATAGG
58493	58R	CAAGCTCGTATTTTATCTTTGTTACC
58549	58.549R	GACGCGTGAATAGATCAACCTTAAAACAAC
58917	58_917F	TCTCGACGTGAATCGTATGTTTGCAAC
59096	59.096F	CCCTTGGCCATGAACCTCCTTTGG
59713	59.713R	ATGGTTCGCGGTTCTAGCAGGTC
59733	59.733R	GCACCGGTGGCAAGTACTCTATGG
59746	59_746R	ATATCTCTCTGTGGCACCGGTGGC
60405	60_405F	GGTCTCCAAAACCCGATGTCGTAG
60424	60_424R	GACATCGGGTTTTGGAGACCCGC
60637	S7_60637R	ATACCGACGTGGTGATCAGTTGG
60945	60_945R	AGCAGTTTTAACAATAACCGCGGGTC
62010	63.01F (is at 62010)	ACAAAATGTTGAATTGAATCGTACCAGTC
62020	62F	GAATTGAATCGTACCAGTCTCTAC
62020	62_020F	GAATTGAATCGTACCAGTCTCTACTGGGG
62020	62F	GAATTGAATCGTACCAGTCTCTAC
62024	M23_62024F	TGAATCGTACCAGTCTCTACTGGG
62354	M23_62354R	GGATGATCCCAATCCGGAATATGAACC
62463	62_463R	GGAAGAACTCAACAGGACCCCCTCG
63463	62R	GGCATCTATTATTTTGGCACAAATC
63471	S8_63471F	ACAACAGACCTTGTACACGTAACGG
63538	63_538R	GGGTGGATTTGGTCAGGGGGATGC
63540	63.5R	GTGGGTGGATTTGGTCAGGGGG
64113	64.113F	CGGGAAGTACCGCTTGTGGAACC
64299	64.299R	ACCCGATCCATTTCCGTATTGCTC
64922	64.922F	GGATACGGTCTTGGTTGTTCGTCTCG
65187	65.187R	AGTCTTTTTGCCCTGGTGCATCTC
65900	65.900R	AGGTCTCTTTCTAAGTTTACATTGGTGGC
66388	66F	AGAAGCCATTGCAACTGCCGGAAA
66388	66.3F	AGAAGCCATTGCAACTGCCGGA
66398	66.398F	GCAACTGCCGGAAATACTAGGCCCAC
66424	66.424R	AGTGGGCCTAGTATTTCCGGCAG
66820	S8_66820R	ACACATGTTTCTTGTAGAAATAGAGGGCA
67295	67R	GGAAGTTTGAGCTTAATGCAAATGG
67299	67.29R	AGAAGGAAGTTTGAGCTTAATGCAAATG
67382	67.382F	TCTCACCCACTACGGATCCCATAC
67529	67.529F	GATAATCATCATTTTCAGAAGGTTCGTC
67534	S9_67534F	TCATCATTTTCAGAAGGTTCGTCTTCG
67977	67.977F	TCCATAACAACTGTCGCAGGCAAC
67988	67.988F	TGTCGCAGGCAACCCACAAATG
68014	68.014R	TTTCGCATTTGTGGGTTGCCTGC
68241	68.241R	CCAAAAGGCTAGTGACATTTTAGAACCG
68665	68.665F	TCCTCCTCCTGCGGATTATTCTCATTG

68689	68.689R	ATGAGAATAATCCGCAGGAGGAGG
68745	68.745R	GGTTATATGCTTTCTGAAATCGGTATTGATAGTTCCA
69044	S9_69044R	AGGGGTTTAGAATACAGGTGTGGTTTAAG
69421	S10_69421F	ACCTGGGACGGAAGGATTCGAACC
69482	69.482R	AAGCGGTAAGGCGACGGGTTTTGG
70253	70.253F	TCTTAGCTTTTGTTTGGCAAGCTGCTG
70257	M10_70257F	AGCTTTTGTTTGGCAAGCTGCTG
70261	70 F	TTTGTTTGGCAAGCTGCTGTAAG
70511	S10_70511R	GGTATTGGTGGTGGACTCCAATCG
70511	M10_70511R	GGTATTGGTGGTGGACTCCAATCG
70815	70.815R	TTCACGTCCAGGATTACGTCCTGG
71002	71R	CGAATCCCTCTCTTTCCG
71041	71.041F	CCGCCGCTTTAGTCCACTCAGC
71062	71.0R	GCTGAGTGGACTAAAGCGGCGG
72027	72.827R	TGGGGAAGCAGAAGCCCTTTTG
72164	S11_72164F	TCCAAGCTAACGATGCGGGTTCG
72834	S11_72834R	CATTCACTGGGGAAGCAGAAGCCC
73176	73 F	CCaACTCTGGAAACGGAaATACCC
73416	73.4F	TACGCTTCGCGACCTGGTGG
73868	73.868F	ACCGGGAGCGGGAGATTCGATTAACC
73938	M24_73938F	GCTAGGGCATTTACAACACGACCC
74164	74R	GGCATTGCTCGTATTtAtGGTCTTG
74177	74.177R	TCAAGTAGGCGACGGCATTGCTCG
74177	M24_74177R	TCAAGTAGGCGACGGCATTGCTC
74513	74.513F	TCGCCCTTTGTTGTTCAAAATGAATCG
75447	75.447F	ACAACTCGCACACACTCCCTTTCC
75570	75.570F	TTCCGGCGGATGGCCAGTAAATG
75573	75.573R	TGCTTCATTTACTGGCCATCCGCC
75992	75.992R	TTATTTAGTCCAACGAGCGCCCC
76015	76.015R	TGGATTCGCCTTCTTCCGTCCC
76290	76.290R	GCGCGACAACCGGAAGCAGAGGAC
76308	76.308F	GCCCTGCAGCTGTACCTTGACC
77424	77.42F	CAATGATGGCCTTCCATGGATTCACC
77428	77F	GATGGCCTTCCATGGATTCACC
77473	77.473R	GCTACCTTAGCTGCGGCTTATATAGGTG
77839	77.839R	TGGTCAGGGGCTCTTTTGCCTTGG
78946	78R	CTGCTCGTTTTTTATCAGAAGTTTGTG
79473	79.4R	TGCACCGTTCACGGTCAAGGC
79479	79.47R	GAATAATGCACCGTTCACGGTCAAGG
79705	79.705R	AGAACGAACTGGGCGTGCCTTG
82013	82 F	CCTGCACGACTTTCCTGATAAG
83013	83R	GAGGATTAATGTCAGATCCACAAGG
86445	86.445F	AGGAGAAACTGATCCAATTCGAAGGTG
86752	86.7F	CCTCGAGCGCCCAAACCTCC
86752	86.75F	CCTCGAGCGCCCAAACCTCCATTTC
86805	86F	CTTCCTTTAAGGGGTTGTTGTG
87780	87.780F	CCCAAAGCGAGTTCACCGCCAAC
87978	S13_87978F1	TTTCCGGCGTGTTCGGCTATAGC
88103	88R	GAGTTCTAATATGCAACGTCAAGC

88515	88.515F	CTAGCAGTTCGCCCCGTCAATCC
88845	88.845F	TTCATTCCAATCAAATGATCCGCAGC
89354	89.354R	GCAAAGCCCTGGTATCTATTATCGTTC
89488	89.488R	TCCGCGGGATTAATTTGGAAAAACAG
89645	S12_89645F	GCCCCGATCAATGAATCTACAAAACCC
89649	89F	CGATCAATGAATCTACAAAACCC
89707	89_707F	ACATTCCTTCATTCCCAACCCCAAGC
89711	89.7F	TCCTTCATTCCCAACCCCAAGCA
89846	89.846F	CTGAATCACATGAAATTTTACCTAACGACG
90065	90.065F	AAATGAATTCAGGATTTGTTTGGCTCC
90069	90.069F	GAATTCAGGATTTGTTTGGCTCCATGAG
90256	90.256F	TGCAGTCCTATTTGTTCGGGACAGC
90281	S12_90281R	TGCTGTCCCGAACAAATAGGACTGC
90422	90.422R	ACAGCAACTGTTAATATATGTCAACCCC
90808	S15_90808F	CGATATGGCCGAGTGGTAAGGCG
90819	90.819F	AGTGGTAAGGCGGGGGACTG
90825	90.825F	AAGGCGGGGGACTGCAAATC
90832	90.832R	CCCGCCTTACCACTCGGCCATATC
90835	90.8R	TCCCCCGCCTTACCACTCGG
90852	90_852F	CCCAGTTCAAATCCGGGTGTCGCC
90941	S14_90941F1	CCAGCACAAGCTAGTGTAGGAATAAGGG
90954	sat_90_954F	GTGTAGGAATAAGGGACTAGTTTGATGC
91182	91.182R	ATTCCCCATCCGAGCTATCC
91187	S13_91187F2	GTCCATTTTAAGTTAGGGAAGGTGTTGAAG
91506	S15_91506R	CCATTTGATTGTCTCCCGATTCGTTC
91616	S14_91616F2	GGATGTTTCGTCAATGGTGTTATGACGG
91619	91.619F	TGTTTCGTCAATGGTGTTATGACGG
91674	91.674R	GGAATCGCTGGTACAGAGTC
91812	91.812F	GTCTTGCTTGGGCTGGTTTAATGGT
91824	91R	GCCCAAGCAAGACTTACTATATCCAT
91828	91.82F	ACCAGCCCAAGCAAGACTTACTATATCC
91839	91.839R	ACTACCATTAAACCAGCCCAAGCAAGAC
91857	91.857F	CCCTTGTAGTATGGGGAAGAAGTGGA
92122	92.122R	TTCCCGGGACAATAAGATGGGAAC
92826	92_826F	AAGGCAGTAGGAACTAGAATGAAGAGTGC
92855	92_855R	TGCACTCTTCATTCTAGTTCCTACTGCC
92873	92.873R	TCTCGCATTTATAGCTACTGCACTC
92879	92.879R	CAATATTCTCGCATTTATAGCTACTGC
92981	92.981R	ACCACTGAATTTACTGACGAAAGATT
93179	S13_93179R	GTTTAAGGTAAAGGTTCGGACGAAGAAAG
93318	93.318R	GGAAGCTGCGGGTTCGAGTC
93329	93_329R	CCTGTCAAGGCGGAAGCTGCGGG
93330	93.330R	CCCTGTCAAGGCGGAAGCTG
93523	S14_93523R	TGCGAATAATCCGTGTCGATCTCGC
93905	94F	AAGTCGAATCCCCGTTGCCTC
94321	sat_94_342R	AAACCGATTCGAATTGAAATCATCTTCATC
94597	95R	TACCACTGAGTTAAAGGGGC
95151	M11A_95151F	TCCACGCAGAAGGGTTTATTCTAAGTTCC
95165	95.165R	ACCCTTCTGCGTGGAGAAGAGGAG

95703	M11_95703F	GAGTAGTCTGTCTGGGGATCTGCTGG
95963	M11_95963R	CTGTCCCTTCTTTTCTACTGGGTTTCG
96214	M11A_96214R	CCCACGGCAAAATAGGCGCAAGG
96215	96.2R	CCCCACGGCAAAATAGGCGC
96352	S16_96352F	TATGGGGTCCTGAAGCACAGGGAG
96718	96_718R	TGAATAGCGCATAGTAGGGCAGCGC
98774	98F	GTTTTCAAGACCGGAGCTATCAAC
98781	98.78F	AGACCGGAGCTATCAACCACTCAGCC
98868	S16_98868R	TCGTATGGTCATGTTCTCTTGAGTGG
99946	100R	AACCCGCGTCTTCTCCTTGG
99947	99.94R	GAACCCGCGTCTTCTCCTTGGCAAG
102995	103F	AAGAATGCCCACGTTGTGGC
103262	M12A_103262F	GCCTGCGCCCATAAGAAATCGCGG
103265	M25_103265F	TGCGCCCATAAGAAATCGCGGAG
103266	M12_103266F	GCGCCCATAAGAAATCGCGGAGC
103521	M25_103512R	CGGTTCCCTTGTGATGGACCTGG
103731	M12_103731R	CCTGGTGCAACAGCAAGCACCAG
103748	M12A_103748R	CACCTGGTACAACGGCCCCTGGTG
104282	104R	GGGGTATTGGTCATGGTATAAGAG
105399	S17_105399F	TGATCCGGAAGTTAATCCGGGGC
105927	S17_105972R	ACAAGCTATAGCCCTTACCCCTGGG
106839	106.8F	AGGGTTTCGTTCGAGTGCCCT
107731	107F	CCATCTCTGTAATAAGTGAATGCC
107979	S18_107979F	CCTACGGCAGGCCTCGAAGTAAG
108113	108R	AACCACTCGGCCATCTCTCCTA
108527	108.527F	GGATTGGCAATTTTTCGCGGGGG
108715	S18_108715R	TGGCTTCGACTATTCCTCAAGCCCG
108788	S19_108788F1	TCGCTTTTCCAGCGATACGAACG
108990	S19_108990F2	ACGAGGCCCTCTGTAACGTGACAT
109384	S19_109384F3	AACCTCTGAGCTAAGCGGGCTCTC
110650	110F	CGAAAtTGGTAGACGCTACG
110670	S19_110670R	CCGTAGCGTCTACCAATTTCGCC
111894	111.894R	AGTTGGATAGGATGGCCTTTGCG
112789	112R	GTTATTGCTATGGGAGCCTG
112898	112.8R	ACGGTCGAGTCCTAGGCAGGC
114808	114F	GAGTCATTGGTTCAAATCCAATAG
114965	M26_114965F	AGATCCTATTGATAGCCTCTACTCGGGTC
115163	115R	GCTAGAATAGGAAATAATGAGATCAC
115178	115.17R	CTGATGGGCGGTTTTGCTAGAATAGG
115189	M26_115189R	GTAACGATGGCTCTGATGGGCGG
116601	116.601F	CCTCTCTTTAGACCATCTGTAGCACTC
116688	116.688F	ATTTCTTGGCCAACAGTATCTCG
117033	117.033R	TGGTTGGGTGGTACCAAGAAAATCG
117587	117.587R	GCAACAACAAGGTCTACTCGACATAAC
117652	117.652R	CGTTGCTTTAGTTTCTGTTTGTGGTGAC
118864	118.8F	ACCTGGTGCCGTAGCGAATCG
118906	118F	ACAAGCTCGTAATGAAGGACGTG
118990	M27_118990F	TGCTGCTTGTGAAGTCTGGAAGG
119446	M27_119446R	TGAGTCAATCCATCCTTTTATTCACGATC

120129	120.129F	CCAAAATCAAAAGAGCGATTGGTCC
120341	119R	TCAAATGATACATAGTGCGATACA
122149	M13_122149F	TAGTTCCGGGTTCGAGTCCCGGG
122158	124F	GTTCGAGTCCCGGGCAAC
122662	M13_122662R	CTGGAGGAGCTGCGATGAAAGCG
123520	123.5F	GCTGCGGTCGAGGCTCCATC
123628	123.628F	ACTCCCCCTTTTACTTTTTGTTAGTAGTC
124027	124.027R	GGCGAATGACGGGAATTGAACCC
