# Supplementary material for: Two Distinct Plastid Genome Configurations and Unprecedented Intraspecies Length Variation in the accD Coding Region in Medicago truncatula
Source: DNA Res. 2014 Mar 17;21(4):417–27. doi: 10.1093/dnares/dsu007 (PMC4131835; doi:10.1093/dnares/dsu007)
Supplement: Supplementary Data [file supp_dsu007_dsu007supp_table2.pdf]

Table S2. PCR amplicons for Illumina library preparation

M. truncatula cv. Jemalong line 2HA	*M. truncatula* cv. Paraggio	*M. truncatula* cv. Borung	*M. truncatula* cv. R108
Primer pairs	Primer pairs	Primer pairs	Primer pairs
118.8F/1.2R	118.8F/1.2R	118.8F/1.2R	1.9F/10.9R
110F/3.6R	110F/3.6R	110F/3.6R	9.555F/11.994R
124F/10.9R	124F/10.9R	124F/10.9R	-
4.4F/10.9R	9.555F/11.994R	9.555F/11.994R	16.867F/18.532R
9.7F/15.5R	-	-	18F/23R
14F/19R	13.777F/16.574R	13.777F/16.574R	22F/26R
18.38F/34.16R	16.806F/18.532R	16.806F/18.532R	25F/30R
33.22F/50.13R	18.38F/34.16R	13.777F/18.532R	29F/34.1R
49F/58R	33.22F, 50.13R	18.38F/34.16R	33.2F/38.3R
53F/71R	49.09F/67.29R	33.22F, 50.13R	37F/42R
70F/88R	62F/74R	49.09F/67.29R	41F/46R
77F/88R	70F/88R	63.01F/79.49R	45F/51R
86.7F/91R	77F/88R	70F/88R	49F/53R
89.7F/96.2R	-	-	53.252F/55.528R
94F/100R	92.826F/96.718R	92.826F/96.718R	54.343F/57.844R
98.78F/115.17R	94F/100R	94F/100R	-
	98.78F/115.17R	98.78F/115.17R	60.405F/63.538R
			62F/71.0R
			66.398F/69.482R
			70F/74R
			73F/78R
			77F/83R
			82F/88R
			86.7F/91R
			89.7F/96.2R
			94F/100R
			98F/104R
			103F/108R
			106.8F/112.8R
			110F/115R
			114F/119R
			118.8F/1.2R
			124F/3.6R
			1.9F/6.4R
