# Supplementary material for: Two Distinct Plastid Genome Configurations and Unprecedented Intraspecies Length Variation in the accD Coding Region in Medicago truncatula
Source: DNA Res. 2014 Mar 17;21(4):417–27. doi: 10.1093/dnares/dsu007 (PMC4131835; doi:10.1093/dnares/dsu007)
Supplement: Supplementary Data [file supp_dsu007_dsu007supp_table3.pdf]

Table S3. Jemalong A17 PCR probes to detect ptDNA inversion in the 2HA and R108 ptDNA

	Primer	Primer sequence	Primer	In R108	2HA/bp	R108/bp
Probe 1	11.852F	GCCATTGAACTTCCCAATCAAATTCCTC	11852	11892	518	518
	12.369R	AGCAATAACCGTAAATGGACCGGAAC	12369	12409		
Probe 2	14 F	CCAGCATTTCTATATCTAGCTCT	13962	56730	551	523
	14R	GATTTCTTATAATTACAACATCTCTATCC	14512	56208		
Probe 3	57F	ATTAGAAACACAAGACAGCCAAT	56890	14483	955	961
	57_844R	TCCCGATGAGCCGAAACCAAAGC	57844	13523		
Probe 4	59.096F	CCCTTGGCCATGAACCTCCTTTGG	59096	58491	638	617
	59.733R	GCACCGGTGGCAAGTACTCTATGG	59733	59107		
