# Supplementary material for: Two Distinct Plastid Genome Configurations and Unprecedented Intraspecies Length Variation in the accD Coding Region in Medicago truncatula
Source: DNA Res. 2014 Mar 17;21(4):417–27. doi: 10.1093/dnares/dsu007 (PMC4131835; doi:10.1093/dnares/dsu007)
Supplement: Supplementary Data [file supp_dsu007_dsu007supp.doc]

**Supplementary information**

**Two Distinct Plastid Genome Configurations and Unprecedented Intraspecies Length Variation in the *accD* Coding Region in *Medicago truncatula***

Csanad Gurdon and Pal Maliga*

Waksman Institute of Microbiology, Rutgers, The State University of New Jersey, 190 Frelinghuysen Road, Piscataway, NJ, 08854-8020, USA

*To whom correspondence should be addressed:

Pal Maliga

Waksman Institute of Microbiology

Rutgers University

190 Frelinghuysen Road

Piscataway, NJ, 08854-8020, USA

Phone: +1-848-445-5329

Fax: +1-732-445-3143

E-mail: [maliga@waksman.rutgers.edu](mailto:maliga@waksman.rutgers.edu)

**Supplementary Figure S1.** The circular plastid genome map of *M. truncatula* R108 line created using the OrganellarGenomeDRAW program[^1^](#_ENREF_1). Genes shown on the outside of the circle are transcribed in the clockwise direction, and those shown in the inside are transcribed in the counterclockwise direction. Black arrows No. 1 and 2 outside the circle point to the inversion breakpoints in the *rps15*-*rpl20* and *ycf1-rps18* intergenic regions. Gene order between the arrows in the 2HA, Borung and Paraggio ptDNAs is in the reverse orientation. Below the map are shown the alignments of imperfect repeat sequences flanking the run of Ts (highlighted in yellow) containing the inversion endpoints in the R108 ptDNA and cognate sequences in 2HA.

**Supplementary Figure S2.** DNA gel blot analysis confirms two stable plastid genome configurations in *M. truncatula* ssp. *tricycla* ptDNA using *Eco*RV polymorphic sites. (A) Schematic map of 2HA and R108 ptDNA with the position of DNA probes P1-P4. Site of inversion is marked by x. *Eco*RV fragment sizes are given inside circles. (B) Probing *Eco*RV -digested total cellular DNA of four 2HA (H) and four R108 (R) plants with probes P1-P4. (C) Testing ptDNA genome structure in *M. truncatula* ssp*. tricyla* lines in *Eco*RV -digested total cellular DNA using probes P1-P4. The lanes contain DNA of: line 2529, T1; 2624, T2; 761, T3; 1665, T4; GR546, T5; 765, T6; W611366, T7.

**Supplementary Figure S3.** The *ycf1* mVISTA similarity plot of Borung, Paraggio, R108 and angiosperm species *Cicer arietinum* (NC_011163), *Lotus japonicus* (NC_002694), *Nicotiana tabacum* (NC_001879), *Solanum lycopersicum* (NC_007898), *Spinacea* *oleracea* (NC_002282) and *Arabidopsis thaliana* (NC_000932) compared to the *M. truncatula* 2HA line. The window is 100 bp, the consensus width is 100 bp and the consensus identity is 70%.

**Supplementary Table S1.** Primers to amplify the *M. truncatula* plastid genome fragments.

**Supplementary Table S2.** PCR amplicons for Illumina library preparation.

**Supplementary Table S3.** Jemalong A17 PCR probes to detect ptDNA inversion in the 2HA and R108 ptDNA.

1. Lohse, M., Drechsel, O., Kahlau, S. and Bock, R. 2013, OrganellarGenomeDRAW--a suite of tools for generating physical maps of plastid and mitochondrial genomes and visualizing expression data sets. *Nucleic Acids Res.* **41**, W575-581.
