# Supplementary material for: Two Distinct Plastid Genome Configurations and Unprecedented Intraspecies Length Variation in the accD Coding Region in Medicago truncatula
Source: DNA Res. 2014 Mar 17;21(4):417–27. doi: 10.1093/dnares/dsu007 (PMC4131835; doi:10.1093/dnares/dsu007)
Supplement: Supplementary Data [file supp_dsu007_dsu007supp_fig1.pdf]

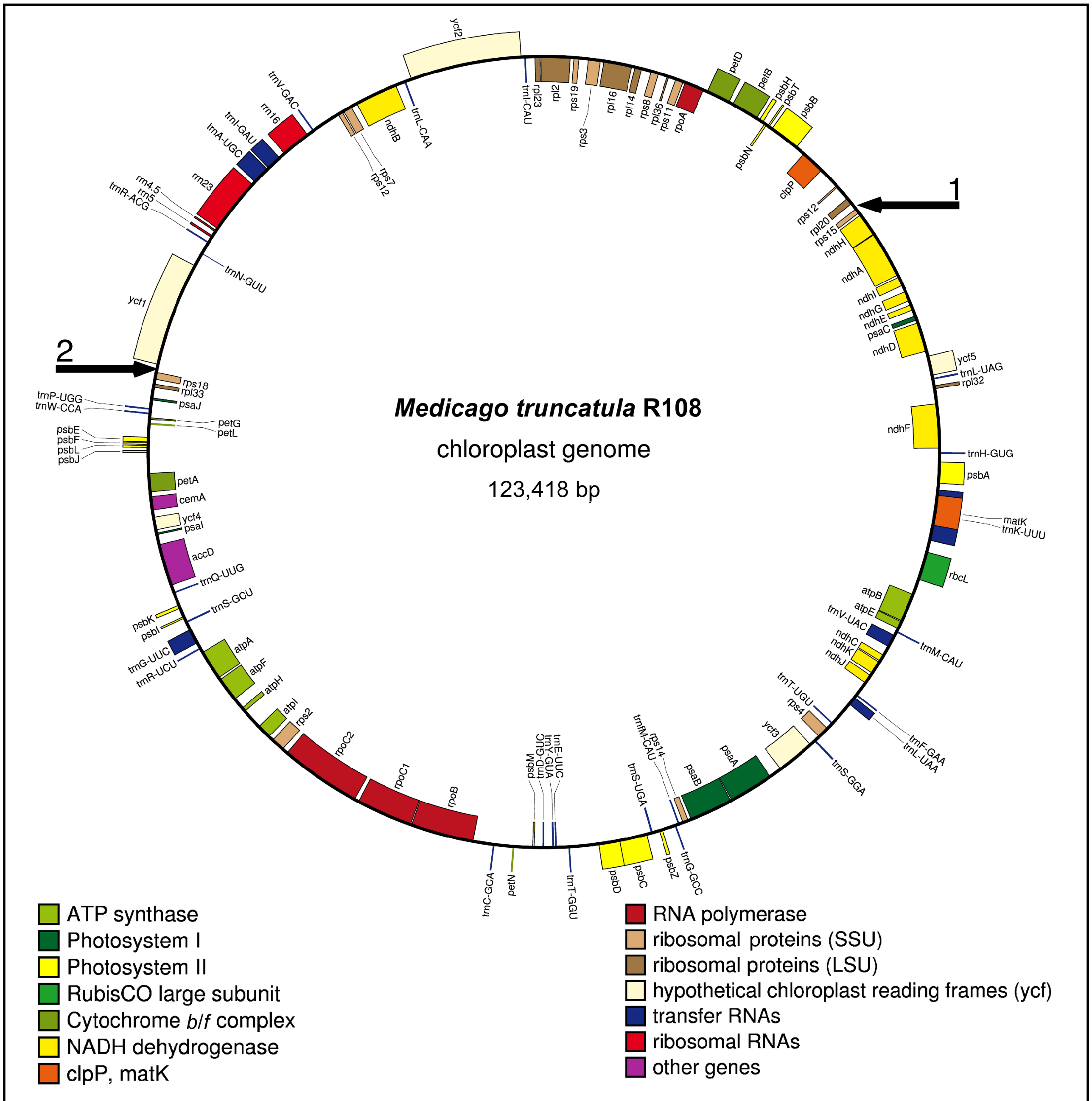


```
2HA 13050-13125     AATTTTTAATTTTGTATATAAAATTATCTTAAAT------TTATTTTTTTTTTTTTTTTTCAAATCAATTATATTATTATA
2HA 58305-58229 c.  TAAATTTCATTTTGAATCGAAACTCAAATTCAATGCAGATTTATATTTATTTTTTTTTTTCGAAAAA-----TACGACAATC
                    :*::***.******:**. ***.* *:.**.***      ****:***:***********  ***:.      **  * :**.

R108 13093-13168    AATTTTTAATTTTGTATATAAAATTATCTTA------AATTTATTTTTTTTTTTTTTTTTCG-AAAAATACGACAATCCTAT
R108 57728-57636 c.TAAATTTCATTTTGAATCGAAACTCAAATTCAATGCAGATTTATATTTATTTTTTTTTTTTCAAATCAATTATATTATTATAT
                    :*::***.******:**. ***.* *:.**.     .******:***:*************. *:.***:. * :** .***
```

Supplementary Figure S1. The circular plastid genome map of *M. truncatula* R108 line created using the OrganellarGenomeDRAW program (Lohse et al. 2013, *Nucleic Acids Res.* **41**, W575-581). Genes shown on the outside of the circle are transcribed in the clockwise direction, and those shown in the inside are transcribed in the counterclockwise direction. Black arrows No. 1 and 2 outside the circle point to the inversion breakpoints in the *rps15-rpl20* and *ycf1-rps18* intergenic regions. Gene order between the arrows in the 2HA, Borung and Paraggio ptDNAs is in the reverse orientation. Below the map are shown the alignments of imperfect repeat sequences flanking the run of Ts (highlighted in yellow) containing the inversion endpoints in the R108 ptDNA and cognate sequences in 2HA.
